# The *Consilia* by Learned Physicians Pietro Andrea Mattioli and Francesco Partini: Dialectic Relations between Doctrine, Empirical Knowledge and Use of the Senses in Sixteenth-century Europe

**DOI:** 10.1093/shm/hkab118

**Published:** 2021-11-29

**Authors:** Alessandra Quaranta

**Keywords:** use of the senses, learned physicians, Pietro Andrea Mattioli, Francesco Partini, *consilia medicinales*

## Abstract

The present article focuses on the medical practice of Pietro Andrea Mattioli from Siena and Francesco Partini from Rovereto (Trent), learned physicians who worked for the Habsburg courts in the second half of the sixteenth century. They paid particular attention to the body signs of disease and described them in detail through the senses of sight, touch, smell and taste. Such a method allowed them to formulate a plausible diagnosis, which concerned not only a general humoral imbalance but also often a specific organ. Furthermore, the empirical data they observed were interpreted in the light of Galenic medicine, a fluid and adaptable system, capable of including relatively new elements. Partini and Mattioli’s medical consultations reveal peculiar aspects of body examination and offer the opportunity both to seize the inventiveness of Galenic medicine and to explore the complex relationship between learned physicians and the written medical tradition.

The present article conducts an enquiry into the sixteenth-century ordinary medical practice, showing how learned physicians arrived at a diagnosis and on which basis they chose their therapeutic approach. In this context, I will investigate the complex dynamics that involved written medical consultations, Hippocratic–Galenic doctrines and empirical methods, and I will emphasise the intricacy and flexibility of medical knowledge in the early-modern period. Scholarly physicians, mainly renowned for their prominent theoretical knowledge, humanistic background and published works, were actually deeply committed to the empirical aspect of their profession. Actual diseases were conceived as questions to be deeply pondered on, and they were often discussed in depth among physicians themselves. Treating individual patients implied a steady never-ending search for a balance between Hippocratic–Galenic doctrines on the one hand and everyday knowledge–practices on the other. The dialectic relationship between learning and practice was reflected in the vocabulary adopted by the physicians in their medical consultations. The latter were imbued with a language implying a significant involvement of the practitioners’ senses in body examination. At the same time, however, many of the terms used referred to the humoral doctrine and its relevant concepts. The importance of sensory information, such as the identification of the bodily signs of disease, and more generally the importance of empirical knowledge, emerges even more in comparison with medieval medical consultations. Empiricism turns out to be particularly remarkable from a methodological point of view too. In this respect, three aspects of the sixteenth-century medical consultations will be underscored: the attempt made by the physicians to exactly locate the pathological processes and the patient’s affected organs, the role of doubt played in the diagnostic process, and the epistemic value of trial and error.

In greater detail, the present contribution will take into account the handwritten *consilia medica* (medical consultations) carried out by two Italian learned physicians, Francesco Partini from Rovereto (1500–69) and Pietro Andrea Mattioli from Siena (1501–78). Both practitioners, highly qualified *medici-physici* trained at the *Studium* of Padua, worked at the service of the imperial Habsburg family at the peak of their career. The *consilium medicum* is a genre of medical literature developed in the late Middle Ages. Produced by a learned physician, it aimed at presenting and discussing the case of a sick person and included therapeutic prescriptions. However, medieval *consilia* did not deal so much with individual cases as diseases: details about the patient were not provided, the temporal order of symptoms was not always recorded and their description was heavily interlarded with references to authorities. Although the *consilium* started from a *casus*, its goal was not describing it per se but redefining it according to medical doctrine.^1^ Conversely, as Mattioli and Partini’s *consilia* clearly show, the attention to individual patients significantly increased in the sixteenth century. Their medical consultations will be firstly set in the broader context of the learned medical practice of the time, which was primarily driven by Giovanni Battista Da Monte’s clinical teaching in Padua. The sixteenth-century medical practice also distinguished itself for both an increasing production of casebooks (serial records of medical notes) and the emergence of two new genres of scholarly medical literature, the collections of *Curationes* and *Observationes*. Both of them paid attention to individual medical cases and bestowed great importance to the observation of signs and symptoms. In this respect, they differed from medieval consultations, in which, by contrast, single cases ended up fading into the background, whereas abounding references to the doctrinal exposition of diseases were included. The handwritten *consilia* presented in this article allow us to better understand how Mattioli and Partini reasoned while facing the complex process of treating a disease, how they combined body examination with medical theories, and how they revived Galenic medicine in an empirical sense.

## Examination of the Patient in the Sixteenth Century

Learned medical practice was given a substantial boost by Giovanni Battista Da Monte, teacher of medical theory and practice in the *Studium* of Padua from 1540 to 1551.^2^ He sharply criticised the Scholastic way of examining the sick, which tended to emphasise both the general features of disease and the theoretical classifications of pathologies according to their description made by previous authors.^3^ Da Monte’s approach was instead based on individual case studies, as it clearly emerges from his *Consultationes medicinales*, gathered and published posthumously by his students.^4^ The work by the Paduan professor offered a model for the description of individual clinical cases based both on the acquisition of the medical history of a patient and on direct observation of the symptomatology and of the objective signs perceived by the physician.^5^ During the examination of his patients, Da Monte made the most of his senses: vital functions were measured through touch, which enabled him to establish the quality of the temperament (hot or cold); by observing the colour of the face, Da Monte could understand which *accidentia* the subject had been through. Sight was used to describe in detail urine and faeces, which gave information about the state of nutritive functions.^6^ A trouble with them might be also indicated by an alteration in the smell of the secretions, or by an irregular pulse, recognised by touch.^7^ Besides examining the patient, Da Monte gathered as much information as possible about the subject: the climatic and atmospheric conditions in which he/she lived, his/her habits (both common and peculiar ones), and the problems he/she was suffering or had suffered from in the past.^8^

A great contribution to the rise of observation in medicine had been provided in the late fifteenth century, when astrologers had sustained regimes of observation, nurturing a growing interest in the practices of observation across various disciplines, including medicine.^9^ The role of observation is testified in the casebooks compiled by the English astrologer Simon Forman in the period 1596–1603. As Lauren Kassell has pointed out, being intellectual products of the early-modern encounter between the physician and the patient, casebooks are able to reveal something useful about healing dynamics. Forman recorded roughly 10,000 consultations, in which, using specific notions of horary astrology, he formulated a judgement about the nature of the disease and the possible therapies for it.^10^

The increasing attention to empirical practice of observation is also refracted in the dissemination of two new genres of medical literature in the second half of the sixteenth century, *Curationes* and *Observationes*, intensively analysed by Gianna Pomata. Although the empirical meaning of *observatio* had all but disappeared in the Middle Ages, these forms of medical writing bear witness to an unprecedented emphasis credited to practice as source of knowledge. The first example of *Curationes* is represented by the *Centuriae* by Amatus Lusitanus, published in instalments between 1551 and 1558. Lusitanus’s main focus of attention was the case narrative, no longer semi-hidden in the doctrinal framework. In this respect, the *Centuriae* differed from the medieval *consilium*, which did not deal as much with a disease, but rather with a sick person. The *Observationes* came out as a specific product of the late-Renaissance humanistic medicine and emphasised the role of the physician as attentive observer of the natural course of disease in each single case.^11^*Curationes* and *Observationes* certainly offer valuable information about how physicians diagnosed and treated patients. As regards medical practice, such published collections do not differ from handwritten ones. The healing methods described within public and private consultations were based on the same foundations, since medical students were taught the same methods at university and during their apprenticeship. However, *Curationes* and *Observationes* slightly differed from handwritten sources in author’s writing style and scope. Firstly, as printed genres of scholar medicine, *Curationes* and *Observationes* were highly structured texts. They had to fulfil literary and rhetorical requirements, which ultimately determined the discourse form. Furthermore, since they were conceived for a wide circulation, they were usually undergoing a long revision before publication. Instead, private notes did not respect theoretical tradition; indeed, they were often unpolished texts.

Secondly, public and private medical notes might have different aims. Written for publication, *Curationes* tended to include the most revealing and interesting cases among those which the physician had seen and aimed at enhancing the reputation of the author as practitioner and scholar. In their instance, expounding rare or unknown cases, *Observationes* reflected ‘extra-ordinary’ practices, rather than a day-by-day healing activity. The choice of such cases could be guided by the author’s desire to highlight his personal diagnostic and therapeutic skills.^12^ By contrast, handwritten case histories did not aim at enhancing the author’s medical skills. Rather, they were drawn up for the benefit of the practitioner himself, who could consult them when, for instance, similar cases turned up. Under this perspective, such medical notes were likely to embrace most of the cases submitted to the physician.

Compared to the amount of published medical writings at our disposal, there is only a small number of handwritten diaries compiled by early-modern physicians containing a significant amount of clinical cases set in chronological order. Among them, we shall mention the medical notes left by Georg Handsch, in which he recorded his practical experiences as a medical student in Padua from 1550 to 1553 and later as an intern of Pietro Andrea Mattioli and Andrea Gallo, both personal physicians to Archduke Ferdinand at the Prague court in the mid-1550s. In the following decade, Handsch was appointed personal physician to Archduke Ferdinand too and later moved to Innsbruck with him. The extensive notes Handsch wrote on his patients offer many new insights into the doctor–patient relationship and the mutual exchange of medical knowledge between doctors and patients at the time. However, Handsch only recorded the cases of those patients that he deemed in some way particularly noteworthy.^13^ Another significant record of learned medical practice in sixteenth-century Europe is represented by the activity of the German physician Georg Palm (1543–91). He practiced medicine as a municipal physician in Nuremberg for 25 years and his notebooks followed the patterns of his day-to-day practice.^14^ Equally important are the three volumes of short notes drafted by Hiob Finzel, a physician active in the small towns of Weimar and Zwickau. He recorded more than 10,000 consultations over almost 25 years, from 1565 to 1589.^15^

As mentioned above, the sixteenth-century medical practice involved the making of the senses by learned physicians, who generally followed Da Monte’s approach.^16^ Sensory examination is a complex issue, and deserves further consideration: to what extent did physicians resort to their senses? Which senses were specifically used and for what purposes?

Firstly, it is to note that sensory perception by physicians was influenced by patients, on whose accounts many practitioners based their diagnoses (for instance, the pain felt or the sensation of hot/cold perceived).^17^ As we will see below, this aspect could even limit the use of the senses by physicians. Secondly, the influence of the deeply felt notion of decency around the body, especially with regard to the female one, prevented physicians from laying hands on the patients’ body. More importantly, many historians have assumed that the prevailing disease theories—and those of humoral pathology in particular—could make a physical examination largely irrelevant.^18^ Actually, as I will show, university-trained practitioners made great efforts to combine an attentive observation of the bodily signs with a doctrinal level. On the one hand, Francesco Partini and Pietro Andrea Mattioli attributed great epistemological value to the sensory signs of disease. By means of sight, smell, taste and touch, they described in detail the outside appearance of their patients (face, eyes, tongue, skin, excretions), as well as the signs of their weakness or strength. Such an accurate observation served the purpose of orientating the diagnosis or confirming it and allowed physicians to identify more precisely the affected body part: not only a general region (such as the abdomen or the chest) but a more circumscribed zone (such as the stomach, the kidneys, the upper respiratory tract or the lungs).

**Fig. 1:**
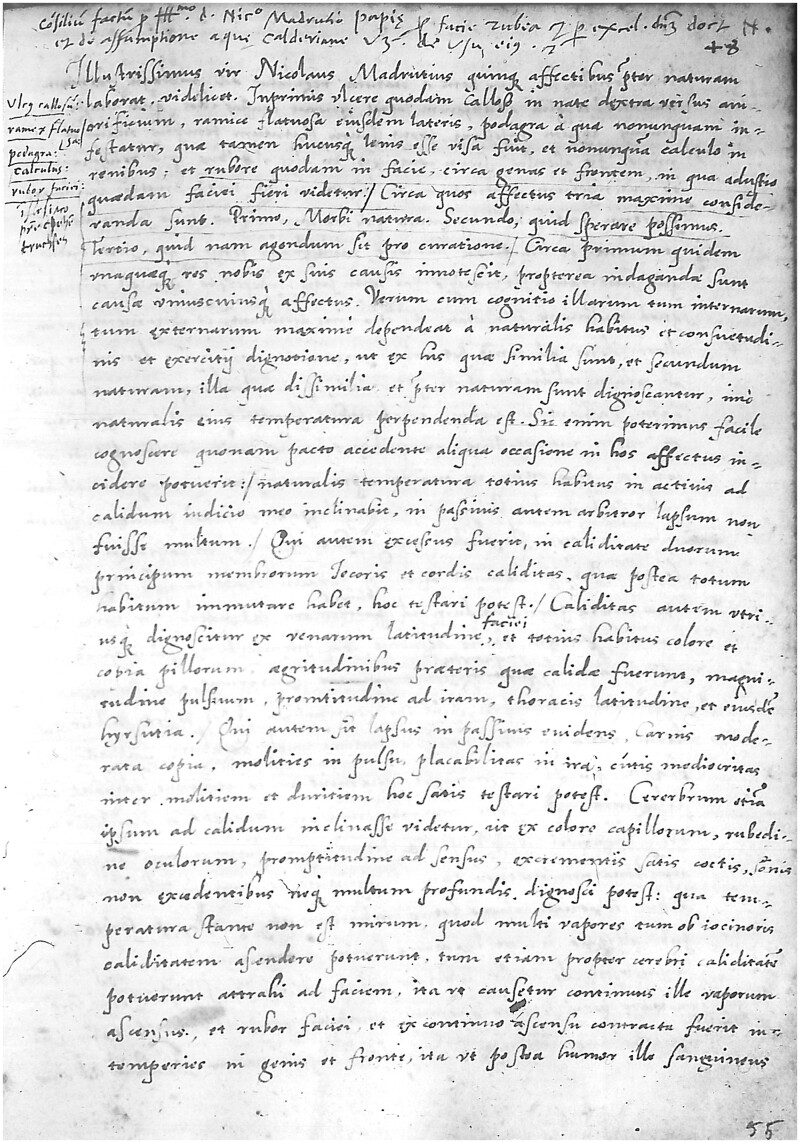
A page extracted from Francesco Partini’s register of medical consultations: Biblioteca Civica Girolamo Tartarotti in Rovereto, section Manoscritti, manuscript 24, ‘Consulti medici e ricette (sec. XVI) di Francesco Partini’, f. 55r

On the other hand, Mattioli and Partini maintained a close relationship with their academic education and linked the data perceived by their senses to the Hippocratic–Galenic theory. The vocabulary adopted by them neatly proves such connections. For instance, some signs, such as noticeable veins on the face, plenty of hair on the chest, the ‘white’ of eyes becoming reddish, hard faeces and a weak pulse were for Partini tell-tale clues to an excessively warm complexion, a term that specifically refers to the Hippocratic–Galenic humoral doctrine.^19^ To the same theoretical framework belonged the catarrh too—the mobile, fluid morbid matter coming from the brain and flowing out of the nose (Figure  1).^20^

## Written Evidence of Mattioli and Partini’s Medical Practice

Pietro Andrea Mattioli and Francesco Partini belonged to the same generation: the former was born in 1501, the latter a year earlier. They both studied in the *Studium* in Padua and obtained the academic qualification of Doctor in *Medicina et Philosophia*.^21^ The two physicians were united by a relationship of mutual respect and affection proven by handwritten letters.^22^ Since they attended the same university presumably at the same time (they were the same age), they might have met each other during their studies. Otherwise, the court of the Prince-Bishopric of Trent might have been the place where they learned about each other. Between 1528 and 1539 Mattioli worked as the personal physician to Prince-Bishop Bernardo Cles.^23^ After graduating in 1531, Partini was appointed town physician by the Government of Rovereto, few kilometres far from Trent.^24^ Afterwards, he attended the court of Cles’s successor, Cristoforo Madruzzo (in office from 1539 to 1572), and cured his older brother Nicolò, commander of the imperial forces.^25^ Furthermore, as we may learn from a letter written by Mattioli to Partini, the ‘Cardinalis Tridentinus’ assigned diverse tasks and honours to the physician from Rovereto.^26^ Since Mattioli wrote in 1554, the ‘Cardinalis’ can be identified with Cristoforo Madruzzo, with whom Mattioli was in contact too.^27^

After Cles’s death, in 1541, Mattioli became town physician in Gorizia, in the county inherited by the Habsburgs at the beginning of the century.^28^ In early 1555 he moved to the Prague court to work as the personal physician to Ferdinand, the second son of Emperor Ferdinand I, Archduke of Austria and Governor of Bohemia.^29^ Later on, in 1562, Emperor Maximilian II granted Mattioli a noble title.^30^

There are many studies about Mattioli and they all agree on the innovative scope of his research activity on vegetable species and medicinal plants, as well as simples and medical drugs. His reputation is mainly connected with his *Discorsi sopra la materia medica* by the Greek naturalist Pedanius Dioscorides, first published in Venice in 1544. This botanical–pharmaceutical work appeared in numerous versions in Italian and Latin and, with the financial support of the Habsburg family, was translated into Czech and German.^31^ Furthermore, Mattioli started developing a concept of *ars medica* in which various scientific currents intertwined: Galenism, Medieval bequests of the Arab culture, empiricism, and the new methods of anatomical and botanical observation, Paracelsianism and alchemy.^32^ In fact, while working for Bernardo Cles, Mattioli tried to introduce various chemical substances of mineral origin into the apothecary shops in Trent for therapeutic purposes, substances that were still unknown to the other Italian apothecaries.^33^

As far as Mattioli’s medical practice is concerned, his hands-on experience is particularly noteworthy, since he has been always invoked by historians as an author of botanical–pharmaceutical treatises. Actually, he also attended to the health of real patients, identifying the specific causes of their diseases and choosing the therapeutic approach that was best suited to fight these causes.

Mattioli left only a few medical consultations. Two of them are contained in the collection *Medicinalium consiliorum liber singularis* edited by Lorenz Scholz in 1610.^34^ Among his handwritten medical texts, there are mostly *receptae*, *secreta* and *antidotaria*, whereas only two can be considered to be *consilia medica* in a strict sense.^35^ Since they are particularly extensive, these two medical consultations well lend themselves to understand how he treated his patients. To this end, Mattioli relied on all his medical and pharmaceutical knowledge. Furthermore, he did not limit himself to mention diagnostic conclusions and the relevant cures but thoroughly enquired into a large range of aspects, such as eating, habits, lifestyle, earlier disease episodes, and body signs and symptoms. Furthermore, he strived to tailor remedies and *regimina sanitatis* specifically according to the constitution and the pathology of the patient.

Partini became the personal *physicus* of Ferdinand I’s son, Maximilian, King of Bohemia and future Emperor in 1557. He managed to obtain this prestigious role thanks to his friendship with Mattioli and the imperial physician Giulio Alessandrini (1506–90).^36^ Like Mattioli, Partini was granted a noble title with an imperial diploma from 1561.^37^

In a collection edited by Georg Hieronymus Welsch, physician and scholar from Augsburg (1624–77), a *consilium* by Francesco Partini is included.^38^ However, the most significant evidence of the latter’s health care activity is certainly represented by the register that he compiled at least from 1536 up to 1567, i.e. 2 years before his death.^39^ Written partly in Latin and partly in vernacular Italian, this notebook contains 80 clinical cases addressed both to the highest members of the aristocracy from Trent and Tyrol and to the members of the imperial family’s *entourage*.

At first reading of Partini’s collection, two stylistic characteristics draw our attention: the writer’s handwriting and the chronological order of consultations. At the beginning of the nineteenth century, Edoardo Benvenuti reported that two people had compiled the codex: Partini and his *scriptor* (or attendant).^40^ In actual fact, by scrutinising the handwriting more in depth, we realise that only one person compiled the register, and that was Partini himself. Most of the *consilia* are written in a regular cursive handwriting, and in the remaining consultations only the main body and title are written in a controlled handwriting, whereas marginal notes are written in a sharp-cornered cursive handwriting, which is also rich in abbreviations. Given these features, it is likely that Partini inserted additional notes many years after he had concluded some of his consultations, but at that time his hand was no longer as stable as his juvenile hand.

Regarding the second feature, the chronological extremes of the register are not determinable due to the lack of explicit time references. Most of the pieces of advice do not chart the date of compilation, and this latter can only rarely be inferred by the content itself of consultations. The most ancient piece of advice with a chronological reference dates back to 1536.^41^ However, even events ascribable to a few years before can be found in the collection. This latter contains advice developed at least until 1567, when Ludovico Madruzzo was elected Prince-Bishop of Trent, to whom Partini dedicated a consultation.^42^

Partini’s collection can be considered to be a typical product of the habit of keeping medical notes in the sixteenth century. As Hannah Murphy has stressed, at least as early as the fourteenth century, physicians kept manuscript records of medical cases, particularly unusual cases.^43^ Thereafter, especially in the second half of the sixteenth century, the practice of keeping medical records—recipes and remedies used, outcome of the cases—further spread among learned physicians.^44^ Manuscripts that detailed series of medical consultations were not unusual for instance among English practitioners, such as the aforementioned Simon Forman.^45^

Nevertheless, with his 80 extensive *consilia*, Partini’s collection turns out to be an exceptional source for historians, at least with regard to the Italian scene. In fact, collections of handwritten medical consultations, which comprise such a large number of cases investigated in depth, have not hitherto come to light. Arguably, only few examples have so far survived, currently buried in archival repositories and not studied yet. As regards the rest of the European continent, I have already noted that some records—Finzel’s practice journal, Forman’s casebooks and Handsch medical notes—have been found out and researched. However, most of Finzel’s notes are short entries; though being a learned man, Forman was not a university-trained physician and his was primarily an astrological practice; as far as Handsch is concerned, he especially recorded those cases that roused his interest.

By contrast, Partini’s register is an extensive casebook with detailed accounts of medical cases. Due to the great amount of consultations it contains and the richness and variety of diseases it deals with, it well lends itself to the investigation into the practical–operational aspect of sixteenth-century health care activity. In this respect, Partini’s collection is for instance different from Hiob Finzel’s casebook. A complete entry of Finzel’s collection included the diagnosis and the medicines prescribed but in most cases, the physician did not reveal the actual symptoms of the patient, the *indicationes curativae* and the effects of the treatments chosen.^46^ Conversely, Partini often provided the reasons why he adopted some remedies and dismissed other ones, and charted the follow-up of his medical interventions. Furthermore, the physician specified both the *regimen sanitatis* the patient had to follow and the medicaments he had to take. The high degree of detail makes us suppose that Partini drafted the consultations not only for the benefit of his patients, but also in order to have well-structured and complete consultations at his disposal in the future.

Partini did not alter the original content of his notes for publishing purposes; therefore, his register is able to attest to some elements that are less probably to be found in published case histories, such as *Curationes* and *Observationes*: diagnostic doubts, failed therapeutic attempts, the questions the physician asked and the related responses, including those that the physician had rejected. Studied as a whole, such elements may both cast light on the specific technical aspects Partini wondered about and clarify how he produced new notions.

Although Partini’s consultations offer the opportunity to investigate further particular aspects, they attest to what was typical of the early-modern practice. There is nothing in Partini’s register that suggests that his practical activity was different from the *practica* of other contemporary learned physicians. Indeed, Partini’s practice was very similar to Da Monte’s. Such similarity between Partini’s and Da Monte’s medical practice does not contradict the differences existing between handwritten and published case histories illustrated above and regarding the author’s scope and style. Though being in print, Da Monte’s *Consultationes* originally consisted in the notes taken by his students for private use. Successively, although such transcripts were probably reworked for publication, they reflected what Da Monte’s pupils had learned from their teacher. Therefore, even the cases that concluded with the death of the patient were worth being written down. In fact, those cases could teach something useful on diagnosis, albeit they did not coincide with successful cures.

Partini’s collection contains two kinds of texts, according to their author: consultations carried out by Partini himself, and recommendations formulated by other physicians on Partini’s request and then transcribed by the latter in his notebook. The plurality of the authors in Partini’s register makes it similar to the notebooks compiled by the above-mentioned Georg Handsch. This latter used to report the opinions of his mentors Mattioli and Gallo related to the clinical cases he was treating.^47^ The consultations produced by Partini’s colleagues—9 out of 80 consultations in all—are either complete *consilia* (structured into the description of symptoms and signs, the recognition of the type of humoral imbalance, diagnosis and prescription of a therapeutic regimen) or replies to a specific question asked by Partini himself. He summoned particularly qualified colleagues: in fact, it was probably the high socio-political rank of his patients that induced Partini to request help from long-experienced physicians, such as Giovanni Battista Da Monte, Giulio Alessandrini and Francesco Frigimelica.^48^

The practice to consult colleagues was connected with the new importance attached to epistolary exchange in the sixteenth century. Great efforts to establish networks of correspondents with whom to share the description of rare cases were being made in this period.^49^ The *consilia* by Da Monte contain, for instance, joint discussions of cases with his colleagues, such as Francesco Frigimelica.^50^ A conspicuous part of private notebooks compiled by Georg Palm included remedies and cures recommended by his colleagues and much of the information he recorded in these notebooks was derived from letters.^51^ Along similar lines, Partini’s medical practice testifies to his firm conviction that it was possible to improve one’s knowledge through discussion and even find more effective therapeutic solutions. Furthermore, his close dialogue with his colleagues, the high frequency with which he consulted them, the promptness of their responses, as well as the meticulous transcription of the advice by Partini are clear signs of the great ferment of medicine in the sixteenth century.

For most of his patients, Partini noted down personal details such as profession and social status; if it was relevant to the diagnosis itself, he added the age of the patient, defining it exactly or approximately. Among the historical figures who relied on Partini’s health care service, it is worth mentioning: Barons Cristoforo and Nicolò Madruzzo;^52^ the latter’s first wife, Helena von Lamberg, Countess of Styria;^53^ Nicolò’s second wife, Geraldina d’Arco;^54^ Prince-Bishop Bernardo Cles, Cristoforo Madruzzo’s predecessor;^55^ Count Sigismondo d’Arco and Vinciguerra d’Arco’s wife, Margaret;^56^ a notable from the County of Flavon;^57^ the daughter of Emperor Ferdinand I, Margaret, Archduchess of Austria; ^58^ a relative of Baron Otto Truchsess von Waldburg.^59^ The latter, one of the most influential members of the Habsburg court, was the dean of the cathedral of Trent, and later Bishop and Cardinal of Augsburg.

## Mattioli and Partini’s *Consilia*

The structure of Mattioli and Partini’s consultations was influenced by the clinical teaching carried out by Da Monte in the 1530s. For instance, in Mattioli’s treatise *De morbi gallici curandi ratione* (1536), the making of sight is documented. The author described the faeces of those who suffered from syphilis as being black and urine as being reddish or leaden and, using touch, he could affirm that those who were infected with *Lues Venera* expectorated dense catarrhal sputum.^60^ In the wake of Da Monte’s teaching, the soul’s accidents had a major role too. After having been asked by Partini on the case of Nicolò Madruzzo’s second wife, Gerardina d’Arco, who suffered from humoral putrefaction and stomach obstruction, Da Monte advised the patient to ‘avoid any emotional upset, especially anger and melancholy’. The woman had to try and force herself to enjoy other people’s company, to be cheerful and keep any worries away.^61^ Emotional states were considered to be equally important by Partini himself and other physicians. Partini thought, for instance, that the loss of his first wife, Helena von Lamberg, had caused Madruzzo to suffer from a serious form of *melancholia*.^62^

Let us now analyse three handwritten medical consultations by Mattioli and Partini. The *consilia* analysed below represent some of the most comprehensive examples of Mattioli and Partini’s ordinary medical practice and can be considered as cases in point of the learned medical practice performed by long-experienced and highly qualified court physicians.

As far as Mattioli’s *consilia* are concerned, the range of choice was limited, since, as mentioned above, only a few *consilia* in a strict sense have survived. Among them, I will present two consultations that can illustrate Mattioli’s medical practice in great detail (cases **a**, **c**). The second one is a joint *consilium*, which Mattioli drafted together with other two physicians. As for Partini, the situation is slightly different. Since he compiled 80 *consilia*, I have been offered the opportunity to look at them as a whole series of consultations and analyse them from a comparative perspective. However, such a task does not correspond to this article’s aim, not to mention that it would require a separate piece of research. Rather, I will focus only on one of Partini’s consultations (case **b**), which is however one of his most extensive *consilia* in terms of body examination and hence allows us to study Partini’s diagnostic process better than the other ones.

**(a)** Mattioli took care of a boy who had kidney stones, the Bohemian Bohuslav.^63^ At the end of September 1560, the young patient started suffering from nausea, followed by vomit, and feeling acute pain in his kidney area.^64^ His stomach having stiffened (‘adstrictum’), he was given an enema (‘clysma’) but, after expelling hardened faeces, Bohuslav was no longer able to urinate. Thus, in order to extract the urine, Mattioli decided to apply a vesical catheter, which was inserted by a surgeon. The extracted urine was aqueous and characterised by ‘whitish filaments and tiny corpuscles’.^65^ From then on, for 8 days, Bohuslav only managed to do a few drops of urine without any traces of blood.^66^ After the eighth day and after taking a diuretic medicine, around midnight he finally managed to urinate.^67^ Then, following another sensory examination, his urine appeared cloudy and oily, with pale sand and small fragments visible in it.^68^ The diachronic perspective adopted by Mattioli immediately stands out, whereas generally medieval consultations included neither references to the development of the ongoing pathology nor a clinical follow-up after the patient had complied with the doctor’s advice.^69^

If we look for instance at the consultations by Taddeo Alderotti (d. 1296), we can recognise further differences.^70^ Alderotti was an authoritative professor in the *Studium* of Bologna and a pioneer in the production of *consilia medica*.^71^ Most of them completely lack the description of symptoms; this may imply that Alderotti did not examine the patients, and expressed his judgement based on the observations made by a colleague (*consilium in absentia*). As Nancy Siraisi has stressed, the primary purpose of Alderotti’s consultations was not to record observations but to recommend treatment. Many of his *consilia* contain no detailed description of the patient’s condition and no account of the process through which the diagnosis was made. Thus, out of Taddeo’s 185 *consilia*, over 100 are simply brief medicinal recipes. Among the remainder, only half describes the patient’s symptoms and often only in extremely laconic terms. Furthermore, their consultations did not deal with day-by-day records of the progress of the disease.^72^ By contrast, the observation of the symptoms over time and the monitoring of their potential evolution provided Mattioli with further elements in order to formulate a diagnosis.

After having observed the symptoms scrupulously, Mattioli supposed that Bohuslav’s difficulty in urinating was not due to a problem in urinary bladder but to a kidney disorder.^73^ He indicated not only the general body region affected but a specific organ. This does not appear to be a minor aspect, since Galen’s treatises, which most physicians relied on in the sixteenth century, provided no secure and consistent basis to be able to distinguish between the symptom and the disease, to differentiate one *affectus* of the body from another, or to relate particular conditions of the body to particular causes.^74^

According to the physician from Siena, fatty material built up in the kidneys because their capacity to filter and expel it was affected by overheating. The violent heat present in the kidneys attracted the raw, slimy material contained in the kidney blood, agglutinated it, and ‘roasted’ it, transforming it into sand and stones.^75^ Bohuslav’s kidneys might have been naturally inclined to a warm quality or the heat might have been caused by external factors, such as excessive physical activity, fatigue due to horse riding, summer heat, or the consumption of food and medicines that overheated the body.^76^ In turn, the toxic, fatty material, which could cause the formation of stones, was produced by the ingestion of certain foods.^77^

In order to validate his diagnosis, Mattioli used the empirical data he had gathered. In fact, he observed that, once the stones had been expelled through the urine, this latter became clear again, and the boy did not suffer from kidney pain anymore. Instead, Mattioli believed that, if the bladder had been the affected organ, the patient’s urine would have remained cloudy and sandy, and the kidney pain would have continued.^78^ It is apparent that the empirical data stimulated Mattioli to reason. Even though Mattioli certainly bore in mind the opinion of ancient and medieval authors on the ailment in question, he regarded the empirical data as sufficient to validate his hypothesis. Conversely, in the consultations written about a century prior to Mattioli’s ones, signs and symptoms perceived by the physician’s senses did not have a fully epistemological value before they were corroborated by precise quotes from ancient authors.^79^ For instance, Bartolomeo da Montagnana, one of the most famous fifteenth-century professors of medicine, and author of a collection of *consilia* produced between 1428 and 1448 and edited posthumously in 1497, tended to turn doctrinal digression into short *tractatus* on a specific topic.^80^ When formulating his diagnosis, he often extended the amount of doctrinal information provided about the illness in question much further than necessary.^81^

It was precisely the analysis of the external appearance of Bohuslav’s urines that allowed Mattioli to formulate an accurate diagnosis. Uroscopy had already become indisputably the most important diagnostic method in the late Middle Ages. It seemed to be able to provide an explanation for pathological processes, which were hidden within the body and not visible to the human eye. In the *De urinis* by Gilles de Corbeil (1140–1214), the palest urine betokened lack of digestion, and therefore of heat, whereas the darkest urine denoted excessive burning of the humours. Thick or thin consistency indicated relative moisture or dryness.^82^ Furthermore, what floated in or settled outside the urine (bubbles, grit, cloudiness, foam, pus, grease, blood, sand, hair, bran, lumps, scales, sperm, sediment) was for Gilles of crucial significance.^83^ Uroscopy was usually adopted to diagnose a vast range of maladies later on too. For instance, Michael Stolberg analysed a certain number of German-speaking patients suffering from different ailments, who sent a sample of their urine to a physician and expected him to formulate a diagnosis based on the analysis of the samples received.^84^ Specific diseases or pathological changes could be identified through uroscopy, which was an invaluable basis for therapy too.^85^ In his instance, Mattioli observed the presence of fragments and sand in Bohuslav’s urine; however, he applied a specific declination of uroscopy, since the urine’s aspect was seen not only as a tell-tale sign of the body’s health but also as a specific symptom closely connected with the organs in charge of the production of the urine (the kidneys).

As far as the therapies prescribed are concerned, Mattioli recommended a lifestyle completely in line with his diagnosis: although physical exercise was usually recommended to overweight patients, Bohuslav had to avoid it, since it could overheat his kidneys even more. Mattioli also prescribed a diet that was adequate for obese bodies: Bohuslav should eat whole grain bread or bread made by millet, since they ‘corpus exicant extenuantque’ (‘dry the body and make it thin’), whereas white bread was prohibited. Eventually, the patient was advised to eat the meat of small wild quadruped animals and volatiles, since they were ‘wetter’.^86^

**(b)** Francesco Partini drafted a *consilium* for Margaret, Emperor Ferdinand I’s daughter. Together with her sisters Helena and Magdalena, Margaret promoted the *Damenstift* foundation of Hall (near Innsbruck), a religious education centre for noblewomen. Margaret, who was very ill, died in 1567 aged only 31 years old.^87^ The *consilium* has no date. However, since Partini became imperial physician in 1557, we can assume that the text was not written before that year.

The woman suffered from acrimonious catarrh whose formation, in Partini’s view, was favoured by her physical complexion, warm and moist. All body parts tended to be hot, and at a certain point, the heat had started to increase due to the overheating of the liver. The heat, in turn, was produced by the fatty foods eaten by the lady and typical of the Tyrol area. The vapour coming from the warm liver had then reached the head, which, being hot, had produced more warm vapour, which was causing the formation of catarrh (‘pituita’). An excessive amount of this humour in the brain, combined with the heat, could not be entirely retained by the head, and therefore reached the respiratory tract, causing violent cough.^88^ Furthermore, the pituitous humour would reach the stomach and, being altered, superabundant and mingled with yellow bile (‘cholera’), it would cause humour and food vomit.^89^ The patient expectorated humours and blood, which, according to Partini, came from the chest. The blood was caused by the erosion of the respiratory tract, which was in turn damaged by the biting humours.^90^ Due to the presence of blood sputum, the physician feared that it could be consumption (‘tabes’).^91^ In particular, there was the risk that the disease might degenerate into that kind of disease called ‘Ptissia’,^92^ a term that indicated a pathology affecting the lungs.

While writing the consultation, Partini wondered what the exact origin of the blood could be: if the blood had come from the trachea, the woman would have felt pain in that area, and a smaller quantity of blood would have been expectorated. But Margaret did not complain about chest pain, nor did she expectorate a small amount of catarrh. Hence, the physician assumed that the blood had invaded her lungs, but he did not have concrete evidence to prove it indisputably. The fact that he required more empirical information to validate his hypothesis is important from a methodological point of view, since it means that a sort of ‘demonstration’ was relevant to Partini. Furthermore, Partini attempted to identify a specific organ as being responsible for the expectorated blood, whereas, in a similar case related to the ‘sputum sanguinis ex pectore vel pulmone’, and treated by Taddeo Alderotti, the origin of the pathology was only approximately identified. Alderotti stated that blood could come either from the lungs or from the respiratory tract,^93^ but he did not give any importance to this question and did not say any other word on it. A methodological difference also emerges between Partini’s approach and Antonio Cermisone’s one, another authoritative writer of *consilia medica*, and professor of medicine in the *Studium* of Padua from 1413 up to 1441, the year of his death.^94^ Cermisone was active as a physician more than a century after Alderotti, and a century before Partini. Cermisone was not concerned with the origin of the pathology. At the beginning of one of his consultations, written for a patient suffering from a headache, the physician stated that the migraine could be engendered by the stomach’s vapours and/or the excessive heat of the kidneys, which went up towards the head and penetrated it causing pain. Further on in the text, Cermisone seemed to change his mind, stating that the altered spleen could also be the cause of the disease.^95^ It is apparent that he did not consider the causes of the disease to be relevant to the diagnosis and therapy.

In Partini’s consultation on Margaret, the description of the symptoms was very detailed, as in Mattioli’s consultation analysed above. The physician examined thoroughly the catarrh’s outside appearance, texture, smell, and taste. Mucus was ‘superabundant’, as established by his sight organs; as far as texture is concerned, catarrh was ‘slimy, wet’—characteristics that could be recognised by touch. Once combined with bile, catarrh would become both salty (‘salsus’), an adjective that refers to the sense of taste, and acrimonious (‘acris’), which usually referred to a biting humour, capable of eroding an artery.^96^ The smell of catarrh, ‘nauseating’, is mentioned several times in the text.^97^ The catarrh’s reaction to mechanical and thermodynamic stress also contributed to revealing its nature: the woman’s sputum, ‘malignant’, ‘unnatural’, ‘altered’, ‘of a bad, blackish colour’, was similar to a ‘conglomeration that swelled up’; it accumulated rather than dissolve; in water, it deposited on the bottom; if thrown over burning coal, it would let out a bad smell.^98^ The same characteristics were recalled later on in the text: ‘Sputum was of a nasty colour, globular, and the eye would shun its sight.’^99^ As in Mattioli’s consultation, Partini continued his direct observation even after administering therapies. In fact, at the end of the *consilium*, the physician pointed out that expectorant syrups had to be taken ‘until a good digestion would appear in the urine’,^100^ that is to say until the urine was pale and clear again, meaning that beverages had been well digested and assimilated by the body. With regard to the detailed description of the symptoms and signs, we have to point out another difference with the above-cited *consilium* written by Alderotti for a patient affected by ‘sputum sanguinis ex pectore vel pulmone’. There was no description of the catarrh and blood in the text by the Bolognese professor, and any reference to their potential evolution was passed over in silence.

Such an accurate description of the body in Partini’s consultation made a great number of elements available to the physician, which could be used in his discussion with his colleagues. For instance, with the data he gathered, Partini could contradict the opinion of Pietro Merenda from Brescia, a physician for the Emperor’s daughters in Innsbruck from 1537 to 1558.^101^ Merenda claimed that there was no inflammation of the respiratory tract,^102^ whereas the quantity and aspect of the blood led Partini to suppose that Margaret’s lungs were inflamed. Furthermore, the observation of symptoms and signs oriented Partini to the prescription of a more suitable therapy for the woman’s clinical conditions, thereby making the two phases, body examination on the one hand and prescription of therapy on the other, much more closely connected. The elements gathered through sensory experience convinced him of the need to purge the entire body: firstly, it was necessary to purify the head, from which the catarrh derived, then the stomach and chest, which received the pituitous mucus.^103^ By contrast, in his consultations, Montagnana declared that no precise identification of the causes was required to choose the proper medical remedy. From this approach, a divergence between the diagnostic section and the therapeutic one arose.^104^

The patient was prescribed decoctions and chest-purging syrups, which were able to dissolve the acrimonious mucus and bilious humour. If the woman had not refused to take them, Partini would have also given her *pillulae aureae* and *pillulae cochiae*.^105^ This implies that Partini paid attention to the patient’s needs and preferences. *Aureae* pills, made with aloe vera, rose, saffron, mastic, vitriol and colocynth, purged the head and reduced the stomach and intestine’s flatulence. Likewise, *cochiae* pills purified the head and stomach from choleric and phlegmy humours, freeing them from pain.^106^ The physician also prescribed *eclygmata* and *masticatoria*.^107^*Eclygmata*, liquorice-based pills to be kept under the tongue until completely dissolved, were able to make the pituitous humour thinner.^108^ They were usually given to those who had short breath, breathing difficulties and persisting cough.^109^*Masticatoria* helped to evacuate excess humours,^110^ and consisted of mastic, stavesacre, nutmeg and *polypodium* root.^111^ After the treatment, the lady managed to expel the mucus more easily, and, as Partini pointed out, the mucus then appeared ‘more clear and balanced’.^112^ Furthermore, she could sleep more easily and gained weight.^113^ Partini also left open the possibility for the patient to bath in the Caldiero waters (Verona), which, being rich in iron, could cool down her liver and reinvigorate her stomach. Furthermore, he asked some colleagues, whose names were not mentioned, whether it was suitable to administer a decoction of sarsaparilla in order to dissolve the pungent catarrh.^114^ Likewise, after thoroughly reasoning on the origin of the disease, he also thought about the most appropriate treatment, by exploring new therapeutic possibilities.

The way Alderotti established the therapy for his patient suffering from spittle of blood from the chest turned out to be completely different. In his advice, the physician reported the therapy, which was recommended by the medical theory for the pathology in question,^115^ and a clarification of the *vitae regimen*, structured in the ‘Six Non-Natural Things’ (*sex res non naturales*: climatic and atmospheric conditions, diet, exercise, sleep–wake rhythm, bowel evacuation and filling, emotional states), ensued. Thereafter, Alderotti suggested a treatment external to the body (an unguent).^116^ In another consultation by Alderotti related to a patient with catarrh, a long exposition of the relevant therapy is to be found according to the Aristotelian method of classification into general cases and subcases:

Cura autem per medicinas est huiusmodi, nam medicinarum quedam assumuntur interius et quedam applicantur exterius. Interius vero assumptarum quedam sunt evacuantes et quedam alterantes et confortantes, inter quas primo prosequendum est de evacuantibus. Est autem evacuatio duplex: una quidem universalis, alia particularis.^117^

And later in the text: ‘After considering purgative medicines, let us look at medicines which alter and soothe’.^118^

**(c)** The third clinical case studied here concerns Archduke Ferdinand of Habsburg, whom Mattioli took care of. The physician from Siena did not deal with this case on his own, but in collaboration with his peer Giulio Alessandrini, already mentioned, and with a certain ‘Dominus Doctor Aiperger’.^119^ The latter is to be identified with Christophorus Heyperger from Vienna, who took a degree in Medicine from the University of Tübingen in 1554, where he had matriculated the previous year at the age of 22 years.^120^ It is possible that Christophorus contributed to treating the Archduke as Mattioli and Alessandrini’s intern. It is also likely that Christophorus was one of Leopold Heyperger’s relatives, manager of the imperial *Kunstkammer* (d. 1560).

Mattioli, Alessandrini and Heyperger’s joint *consilium* is not dated but was certainly written after Mattioli’s arrival in Prague, dated February 1555. Furthermore, it is to be found in a handwritten codex containing another *consilium*, also addressed to Archduke Ferdinand, written by Renato Brasavola from Ferrara in Innsbruck on 14 February 1554.^121^ Renato was the son of the celebrated physician Antonio Musa and enjoyed a position as Professor of ‘Logica ordinaria’ at the *Studium* of Ferrara.^122^ His father Antonio had taught at the same University in the 1530s too and served as personal physician to Pope Paulus III in the following decade.^123^

The two *consilia* do not seem to be directly linked, that is Alessandrini, Mattioli and Heyperger did not seem to have answered a specific consultation request from Brasavola, or vice versa. In fact, the physician from Ferrara is not mentioned in the joint *consilium*, nor does Brasavola mention Mattioli, Alessandrini and Heyperger. However, both the joint *consilium* and Brasavola’s *consilium* focus on Ferdinand’s headache.

Ferdinand suffered from dizziness (‘vertigines’) and nerve obstruction (‘nervorum obstructiones’), which caused forearm numbness (‘stupor brachiorum’) and daze (‘imbecillitas capitis’). Ferdinand’s head, which when in good health tended to be warm, would fill up with moist, pituitous humours that were not well digested by the stomach and were not absorbed by his body. Thus, they would go up towards the head in the form of vapour, increasing the head’s phlegm and causing the above-mentioned problems.^124^ These problems were worsened by the cold, moist air the Archduke was often exposed to: by penetrating the head, the air would cause catarrh, headache, dizziness, sleepiness and/or limb paralysis and pains in the joints.^125^ Mattioli explained in detail the characteristics of the air of Innsbruck:

Est praeterea huius loci Aer valde inaequalis. Namque saepe accidit, ut una, et eadem die, immo aliquando una et eadem hora incalescat, et rifrigescat maxime. Id quod non solum corpora, et membra malis obnoxia destruit, sed, et sana, et robusta non parum afficit.^126^

Based on this consideration, the patient was supposed to avoid the rainy, snowy weather of Innsbruck, at least in winter.^127^ Furthermore, he needed to avoid places around rivers and lakes, where the air was too damp and altered the body and the brain.^128^ Later on in his consultation, Mattioli paid great attention to those behaviours that could prevent the disease from worsening, taking the classical scheme of the ‘Six Non-Natural Things’ into account. This suggestion and other similar ones aimed at preserving a healthy body and implied that disregarding healthy living rules could engender pathological processes.

As regards diet, it was advisable to prefer food that would minimise the production of pituitous, wet, melancholic humour. In order to prevent an excessive amount of phlegm from accumulating in the brain, the patient was advised to avoid eating beef, venison and hare, especially if not well done or if the animals were old. Meats producing wet humours and generating ‘a purulent secretion which could easily rot’ were also banned: pork, bear and suckling boar.^129^ Among spices, radish was banned because it produced smelly burps and affected the head with its sour smell, and was badly digested by the stomach. Parsley, mint, marjoram, thyme, lemon balm and sage could be beneficially used to season dishes.^130^ It is worth mentioning that Mattioli used a rich, descriptive vocabulary, which referred to sensory perception, in order to define spices. Furthermore, it was the sensory acceptance of or repulsion for spices that guided him to decide if they were adequate remedies or not.

As far as medicines are concerned, mastic-based *Assaiaret* pills were prescribed, ‘effective against pain, dizziness, and migraine’. According to Mattioli, these pills caused joy and preserved the energy of the mind.^131^ They were meant to be chewed in order to purify the head from toxic humours. Their basic ingredient was mastic, a substance secreted by the plant *Therebintus lentiscus*.^132^ In addition, Ferdinand had to try and expectorate surplus catarrh as much as possible, taking white hellebore powder.^133^ The roots of this plant were used to evacuate excess humours: by inserting them in the nostrils, they caused violent sneezing.^134^ This effect was caused by the toxicity of the roots, which had a nauseating taste and an equally unpleasant smell.^135^

## Role and Purposes of the Senses

As cursorily mentioned above, at the beginning of their encounters with physician, patients provided basic information about their ailments, illustrated their symptoms, and tried to explain what they felt and observed going on within their bodies.^136^ In turn, physicians paid great attention to the patients’ narratives and to this end, relied on the sense of hearing. After listening to the patients’ reports, they began to conduct a sensory examination, which was primarily meant as the identification and description of sensory signs of disease leading to medical diagnosis. For this purpose, the sense of hearing was only rarely used (physicians might for instance describe the sound of cough), whereas sight seems to have played an overriding role. The visual characteristics of the body, in particular the external appearance of the skin, face, and eyes, mucus, urines and faeces were described in detail. Exactly through such a rich description, Mattioli and Partini were able to sketch out a first diagnostic hypothesis. Albeit less frequently mentioned, taste, touch and smell also contributed to recognising the sensory data of disease. Margaret’s catarrh was for instance described as being respectively pungent, slimy and wet, and stinking. This information alone could not however provide a sufficiently revealing clue to a plausible diagnosis and had to be integrated with the visual features of the catarrhal fluid.

As we have just seen in the case of Ferdinand, smell could also play a major part in therapeutics. In the Galenic tradition, smell was thought to be a kind of vapour coming from the substance of something and able to penetrate the brain, thereby giving it the qualities of the substance itself. Cold smells, like the scent of roses and violets, were good for those who had a warm complexion; instead, those with a cold, moist temperament were to inhale warm smells, like lemon, mint, amber and musk. In his collection of *Consilia* (Venice, 1497), Bartolomeo da Montagnana used dry, warm smells for cold brain complexions, for various types of headaches and catarrh, melancholy and nervous disorders. What is more, it was thought that pleasant smells could have a positive impact on vital, animal and natural spirits, stimulated by the resemblance between the substance the smell came from and the one contained in the spirit itself. Likewise, in his *De saporum et odorum differentiis* (1583), Juan Bravo, professor of Medicine in Salamanca, remarked that fetid smells damaged human spirits.^137^

Touch, taste, smell and sight were all involved in uroscopy. As noted above, this practice was extensively adopted and can be defined as a diagnostic tool in its own right.

Other similar medical instruments, such as the pulse-taking technique, the survey of the warmth of the skin and the exploration of the abdomen, were practiced.^138^ However, Mattioli and Partini hardly ever made reference to such practices or, at most, mentioned them cursorily. This aspect can be explained by the fact that, if the physicians regarded some sensory information as not sufficiently revealing, they did not take a note of it. Taken individually, the aforementioned medical gestures were not probably sufficient to precisely diagnose and needed to be underpinned by other more striking signs, like a ‘nauseating odour’.

At any rate, that Partini and Mattioli made no (or only brief) reference to such gestures does not mean that they did not exercise these practices whatsoever. For instance, as we noted above, among the numerous signs affecting a man suffering from overheating of the liver and the stomach, Partini numbered a ‘weak pulse’. This sign necessarily entails that he applied the pulse-taking technique.

As far as the palpation of the body is concerned, it played a smaller role in early-modern medicine than in the late nineteenth; however, it was more widespread than historians have so far assumed.^139^ In Mattioli’s consultation for Bohuslav, for instance, it is said that the patient’s belly was stiffened, which implies that the physician palped it.

Touch was also connected with surgical interventions, which, in turn, required the ability to precisely use the hands. In particular, since Bohuslav was in trouble urinating, Mattioli got a surgeon to insert a catheter into the boy’s urinary canal. Furthermore, since Bohuslav’s abdomen was stiffened, the boy was administered an enema. If in the *consilium* it is clearly stated that it was the surgeon to insert the catheter in the urinary canal, conversely, it is not said whether Mattioli or the surgeon injected the liquid in Bohuslav’s rectum. Therefore, we cannot exclude that Mattioli himself administered the enema. If that had been the case, it would testify to Mattioli’s acquaintance with surgical interventions. This skill would turn out to be particularly noteworthy, since Mattioli is usually thought of in terms of a scholar in the field of botany and pharmacy. If, by contrast, Mattioli had ordered the surgeon to give the enema, it would imply that the learned physician needed the help of an empirical practitioner. In both cases, we can assume that Mattioli had close professional contacts with surgeons. In fact, the first case allows us to suppose that Mattioli acquired practical notions from a surgeon; the second case proves a form of factual cooperation between Mattioli and a surgeon, further confirmed by the fact that Mattioli also discussed the origin of Bohuslav’s ailments with the surgeon himself.^140^

An ultimate aspect has to be discussed. As briefly mentioned above, the sensory perception by physicians could be influenced by patients. Let us look at Bohuslav’s case. From the consultation, we can drive that the boy said to Mattioli that he felt pain in the kidneys area. The patient’s narrative might induce Mattioli to palpate the body region mentioned by him, although we have no written evidence of that. Subsequently, after administering certain remedies, Mattioli wrote that Bohuslav felt no longer pain: did the physician decide to palpate the kidney area again, exactly to elicit such patient’s response? Unfortunately, the consultation does not provide precise information about that either. However, that Mattioli wrote down Bohuslav’s perception implies that he considered it as relevant and, for this reason, it could influence the physician’s sensory survey.

Furthermore, the patients’ reports could often be incomplete due to single or concurrent factors: embarrassment caused by disease, reticence in admitting intimate details concerning their bodies, fear of diagnosis and fear of not recovering. Omissive or even misleading patients’ declarations could influence the body examination made by physicians and ultimately their diagnostic conclusions. More importantly, the sufferer’s report did not imply *per se* the existence of a pathology: it was the physician who, with his interpretation, gave form and attributed significance to the patient’s sensations. Even if the patient exposed a clear idea of what was happening, his report was interpreted by the physician’s way of looking at an ill body. For this reason, the key to Galenic interpretation, which physicians relied on, is crucial in our analysis.

## The Learned Physicians’ Relation with Galenic Medicine, a Fluid System of Doctrines

As Hannah Murphy has recently remarked, the *Collegium medicum* of learned municipal physicians founded in Nuremberg in 1592 based its identity on Galenic medicine. The reformation of medicine it undertook was heavily shaped on a conservative Galenic movement, but at the same time, it gave form to the turn to empiricism as a foundation for medical epistemology.^141^ Such an epistemological combination was possible by virtue of the fact that the so-called Galenism was actually a system of doctrines susceptible to embracing relatively new concepts and methods, and exactly such fluidity was one of the peculiar features of Galenic medicine in the early-modern period. Partini and Mattioli’s medical practice was also characterised by both a strong continuity relationship with the past and an attitude to attach great importance to empirical data. What Partini and Mattioli observed and perceived was largely informed by the written medical tradition and, as university-trained physicians, they extensively relied on its theories to make sense of the patients’ diseases. The concepts adopted by both physicians and the terms used to express them prove that the Hippocratic–Galenic humoral theory and pathology, considered as fundamental by sixteenth-century academic medicine, framed the way in which they interpreted the data perceived by their senses.

Partini and Mattioli’s relationship with ancient and medieval *auctoritates* is to be set in the context of a slow and trying evolution, which medicine was undergoing in the sixteenth century. Hippocratic–Galenic knowledge was constantly re-read, re-interpreted and even criticised with regard to particular statements. Medical Humanism certainly stimulated physicians to critically read ancient medical texts. For instance, Galen continued to be translated and commented on over the century.^142^ The intensive study of his works brought physicians to criticise some of his opinions on specific topics. For example, the anatomic discoveries carried out by Andreas Vesalius contradicted Galenic anatomy with regard to various passages. However, as Andrea Carlino has emphasised, the renewal fostered by Vesalius entirely occurred within the humanistic culture which the Flemish anatomist himself belonged to. In fact, the Vesalian revolution was conducted in line with the humanistic model of re-foundation of medical knowledge, anatomy included.^143^

The basic outlines of the humoral theory remained those laid down by Galen even in new medical theories, and the challenges to Galen’s authority often resulted in compromise rather than total victory.^144^ Giovanni Argenterio (d. 1572), one of Galen’s most obstinate critics, did not aim at abolishing the Hippocratic–Galenic humoral theory, nor did he propose an alternative one, which he considered more valid.^145^ Girolamo Fracastoro adopted the concept of ‘contagion seed’ (*seminarium primum*), in order to explain the contagion mechanism of the plague: this conglomeration of particles worked regardless of climatic conditions, but affected a subject whose humoral complexion was similar to it and was ready to receive it. According to this innovative theory, both the ancient concept of sympathy and the humoral theory continued to be taken into serious consideration.^146^ Humorism was in fact anything but an unchanging body of knowledge. By contrast, it was a plastic and adaptable system, able to integrate new elements into medical practice (such as chemical remedies), without altering its theoretical framework.^147^

Galen’s reception, as well as that of any other ancient (or medieval) author, was selective and influenced by the cultural sensitivity of the time. The choice of the texts which early-modern physicians drew on depended on both the overriding medical tendency at a given time and the convictions peculiar to individual practitioners. At least two examples can support such a statement. Andrea Gallo, archiater of Archduke Ferdinand, was the author of a study on the nature, causes and possible therapies for the plague, the *Fascis de peste*.^148^ The author identified the sublunary factors (meteorological, geographical, hygienic factors) as being responsible for the plague. Instead, he disregarded the influences of celestial bodies,^149^ straying from the tendency which, based on Avicenna’s opinion (d. 1037), had been established since the fourteenth century. The Persian physician had proposed an interpretation of the pestilence based on the miasmic theory integrated with celestial phenomena.^150^ As regards the second example, during the seventeenth century, the emphasis on the benefits of exercise on the body decreased in comparison with the recommendations from Antiquity and the Middle Ages. The new ideal of physical activity was connected with an emerging aristocratic culture, which promoted new patterns of genteel life and a new ideal of the male body. In this context, Avicenna’s *Canon* began to be considered as better suited than Galen’s *De sanitate tuenda* to convey the new approach to exercise.^151^

Partini and Mattioli's relationship with Galenic medicine was dialectic too. As regards ancient and medieval authorities quoted in their *consilia*, both physicians cited them less frequently than, for instance, Bartolomeo da Montagnana did in the fifteenth century. This latter reduced the cases observed to the doctrinal exposition of diseases and felt the need to check whether the empirical data observed by him actually complied with the general causes provided by the doctrinal frame of reference. It seems that such an approach was applied until the 1520s, when Da Monte’s teachers were used to formulating the diagnosis before recognising all objective signs; after diagnosing, they listed the symptoms of the disease as Avicenna had described them, whether they were present or not.^152^

Conversely, Partini and Mattioli were totally engaged in the study of individual cases and such case-oriented attention resulted in a smaller number of authorities’ quotations. Apparently, both physicians did not tend to verify that everything they had found out through their sensory perception was exactly mentioned in medical literature too. Rather, in the texts by *auctoritates*, they sought for a key to interpret the empirical data observed. Therefore, the minor frequency of their quotations did not correspond as much to a writing convention different from the medieval one, but rather to a diverse way to look at ancient authors.

Nevertheless, Mattioli and Partini bore in mind and explicitly quoted them in specific cases, especially if they had to dispel a doubt or endorse a hypothesis. By treating Margaret’s case, Partini disagreed with his colleague Pietro Merenda. The latter excluded an inflammation of the respiratory tracts, whereas Partini bestowed great importance to the blood expectorated, and he supposed it was deriving from lungs. Partini supported his hypothesis with a passage from the Galenic *De locorum affectorum notitia libri sex*, in which it is to read that, when accompanied by grip, blood did not stem from either stomach or the brain, but from the chest.^153^ After recording all Bohuslav’s symptoms he had observed over time, Mattioli assumed that the boy suffered from a pathology affecting the kidneys, not the bladder. Not completely sure of his assumption, however, Mattioli discussed it with the surgeon who had inserted the catheter in the patient; eventually, he found a satisfactory answer in *De locis affectis*. Here, Galen argued that those who were affected by nephritis (‘nephritici’) initially emitted watery urines, which, in the following days, were filled up by fat matter and ultimately by stones. But once the stone had come out of the body, the patient no longer felt pain in the kidneys.^154^

Opinions by ancient and medieval *auctoritates* could be belied too. For instance, according to Mattioli, the *causa efficiens* of Bohuslav’s ‘morbus nephriticus’ was represented by the kidneys’ affected capacity to eliminate fatty material, together with the violent heat they contained, which converted humours into sand.^155^ This version was not in line with Aëtius of Amida’s thinking, according to which the functional cause ‘huiusmodi affectus’ (‘of the disease of this kind’) corresponded to frequent indigestion. In the Byzantine physician’s view, kidney stones developed when the food ingested, which had not been assimilated by the body, formed small stones in the kidneys. Instead, Mattioli believed that the obese constitution of the boy demonstrated *ipso facto* that the corrupted humours deriving from the foods he had eaten were digested and absorbed by the body. The *causa efficiens*, then went on Mattioli, was not indigestion itself but rather kidney overheating.^156^

## Concluding Remarks

Mattioli and Partini dealt with diseases as actual problems, which equally required actual solutions. They had to treat real cases, which were not often embraced by medical literature and paid attention to individuals and their characteristics and needs. For this reason, they relied on all means at their disposal (direct observation, sensory perception, reasoning, dialogue with colleagues and patients), in order to get health improvements. Within such a methodological context, doubts were considered useful too. For instance, Partini discussed in depth the possibility of applying a range of additional remedies to Margaret, such as vesicants (*vescicatoria*), suction cups (*cucurbitulae*), painful bindings (*deligationes*).^157^ Furthermore, he calibrated therapies according to Margaret’s needs, because she had refused *pillulae aureae* and *pillulae cochiae*.

As to Partini and Mattioli’s diagnostic activity, they considered it as an empirical practice rather than an abstract, philosophical one. This aspect can be traced back to Da Monte’s teaching and work, which constituted a crucial common frame for learned physicians of the time and especially for those who trained at Padua. Mattioli and Partini were very concerned with the localisation of pathology processes. Other than a general humoral imbalance of the body, single organs like the liver or the brain were often specifically indicated by them as responsible for the ailments the patient suffered from. Moreover, they tried to demonstrate the existence of connections between signs and symptoms on the one hand, and the origin of the pathological process on the other, and they managed to identify the affected body region more precisely than their medieval predecessors had done. For instance, Bohuslav’s urinary ailment was traced back not to the bladder, but to the kidneys. What at first seemed to be a disease affecting Margaret’s respiratory tract was then identified more precisely as a lung pathology. In fact, as noted above, Partini attributed greater importance to the patient’s haemorrhagic cough than Merenda had done. Furthermore, he realised that vomit was not the symptom of a stomach disease, but a consequence of ‘ptissia’: as seen above, a part of the acrimonious catarrh had descended from the head into the stomach, causing indigestion. It is also to note that Mattioli and Partini paid attention to the development of the illness over time, and even recorded the effects of the administered remedies.

All that said, the early-modern medical practice was still influenced by the classical medical tradition. This is demonstrated by the fact that the morbid matter of which Mattioli and Partini attempted to identify the nature, source and location corresponded to a typical concept of humorism. Furthermore, Mattioli and Partini assigned great importance to prevention and treatment too, just like Hippocrates, Galen and Avicenna had done. The latter had conceptualised medicine not just as a healing art, but also as the art of well-being and of preserving health. In this context, a key role was played by a healthy lifestyle, which meant educating patients to take the necessary precautions to pursue a long and healthy life. The method for conducting a healthy life was structured around the ‘Six Non-Natural Things’, to which, as seen above, Partini and Mattioli attached great importance. In fact, their consultations clearly expressed the idea that changes within the body were caused by environmental factors such as air and food, and behavioural factors, like exercise. Furthermore, the impact of one’s emotional life on health, already regarded by Galen as a key component for a healthy lifestyle, was a theme of great concern for both Mattioli and Partini. In such a context of continuity, the therapies they administered were not different from medieval ones. For instance, *cochiae* pills were prescribed both by Alderotti and, almost three centuries later, by Partini.^158^

However, Mattioli and Partini’s consultations have different characteristics from the medieval ones. As far as the structure of fifteenth-century *consilia* is concerned, it was entirely based on logical arguments.^159^ Most of them prescribed a general regimen of healthy living, recommending exercise, moderate food and drink intake, rest and regular purgation, regardless of the diagnosis.^160^ In some medieval consultations, sensory perception data were mentioned but they were immediately integrated into a network of general pathologies and causes, which, in turn, justified them and provided them with the status of signs-symptoms or proof-testimonies.^161^ By contrast, in Partini and Mattioli’s consultations, great attention was paid to individual patients. Their habits, external appearance, symptoms and excrements were described in detail. The high level of detail could depend on a socio-psychological factor: the physicians’ necessity to meet the expectancies of their high-ranking patients, in order not to lose the prestigious professional position they managed to reach. However, such accurate descriptions can also be explained with the firm physicians’ conviction that direct observation was indispensable for making an accurate diagnosis and even for finding an appropriate therapy.

In closing, medical practice required a great ability to find a balance between theoretical and empirical levels to fulfil a factual purpose, the patient’s recovery. Empirical data were supposed to have epistemological value themselves but were interpreted within a context in which the study of the ancient and medieval medical literature remained open to new elements and interpretations. In this context, data driving from observation could be used to better comprehend, further explore and even enrich Galenic doctrines. As flexible and adaptable, these latter were steadily being discussed, and the diagnostic process based on empirical observation could urge physicians to make new inventive interpretations of them.

Partini and Mattioli’s consultations reveal their steady will to investigate, study in depth and call into question the written medical tradition. This tendency contributed to fertilising a terrain on which other medical disciplines, such as physiology and chemistry, thereafter developed. From this perspective, ‘Galenism’ could be renewed thanks to the use of the senses too. If we read Partini and Mattioli’s advice in their eyes, we will be able to understand how the physicians of the time treated diseases: they acted with determination before diseases, carefully pondering on remedies; their therapeutic choices were adequate (though not always effective) and had their reason for being.

## Footnotes

1 Gianna Pomata, ‘*Praxis historialis:* The Uses of *Historia* in Early Modern Medicine’, in Gianna Pomata and Nancy G. Siraisi, eds, *Historia. Empiricism and Erudition in Early Modern Europe* (Cambridge-London: MIT Press, 2005), 105–46, 122–23.

2 Jerome J. Bylebyl, ‘The Manifest and the Hidden in the Renaissance Clinic’, in William Frederick Bynum and Roy Porter, eds, *Medicine and the Five Senses* (Cambridge: Cambridge University Press, 1993), 40–60, 40.

3 Pomata, ‘*Praxis historialis’*, 128.

4 Jerome J. Bylebyl, ‘The School of Padua: Humanistic Medicine in the Sixteenth Century’, in Charles Webster, ed, *Health, Medicine and Mortality in the Sixteenth Century* (Cambridge: Cambridge University Press, 1979), 335–70, 346–47.

5 Pomata, ‘*Praxis historialis*’, 128–29; Ian Maclean, ‘The Medical Republic of Letters before the Thirty Years War’, *Intellectual History Review*, 2008, 18, 15–30, 20; see also Michael Stolberg, ‘Bedside Teaching and the Acquisition of Practical Skills in Mid-Sixteenth-Century Padua’, *Journal of the History of Medicine and Allied Sciences*, 2013, 69, 633–61, 645.

6 Bylebyl, ‘The Manifest’, 46–7.

7 Marylin Nicoud, ‘Salute, malattia e guarigione. Concezioni dei medici e punti di vista dei pazienti’, *Quaderni storici*, 2011, 46, 47–74, 52–3.

8 Bylebyl, ‘The Manifest’, 42. Da Monte’s approach was largely used. In his *Index sanitatis* (1539), the *Stadtphysikus* Philipp Begardi from Worms stated that the patient (but sometimes even his/her family and friends) had to be questioned about his/her lifestyle (diet, sleep–wake rhythm, bath frequency, whether he/she bathed in freshwater or mineral/thermal waters) and his/her job and social class. Robert Jütte, *Ärzte, Heiler und Patienten. Medizinischer Alltag in der frühen Neuzeit* (Munich: Artemis & Winkler, 1991), 108–9.

9 Lauren Kassell, ‘Casebooks in Early Modern England: Medicine, Astrology, and Written Records’, *Bulletin of the History of Medicine*, 2014, 88, 595–625, 602.

10
*Ibid*., 596–99.

11 Gianna Pomata, ‘Sharing Cases: The *Observationes* in Early Modern Medicine’, *Early Science and Medicine*, 2010, 15, 193–236, 199, 203, 205–8, 216.

12 Pomata, ‘Sharing Cases’, 209–10; Pomata, ‘Praxis historialis’, 133; Michael Stolberg, ‘A Sixteenth-century Physician and His Patients: The Practice Journal of Hiob Finzel, 1565-1589’, *Social History of Medicine*, 2019, 32, 221–40, 222.

13 Michael Stolberg, ‘Empiricism in Sixteenth-Century Medical Practice: The Notebooks of Georg Handsch’, *Early Science and Medicine*, 2013, 18, 487–516, 490; Michael Stolberg, *‘You Have No Good Blood in Your Body.* Oral Communication in Sixteenth-Century Physicians’ Medical Practice’, *Medical History*, 2015, 59, 63–82, 64–5.

14 Hannah Murphy, ‘Common Places and Private Spaces: Libraries, Record-Keeping and Orders of Information in Sixteenth-Century Medicine’, *Past and Present*, 2016, 230, 253–68, 261–62.

15 Stolberg, ‘A Sixteenth-century Physician’, 223–24.

16 On the history of the senses in general see at least: Martin Jay, ‘AHR Forum: The Senses in History. In the Realm of the Senses: An Introduction’, *American Historical Review*, 2011, 2, 307–15; Mark Michael Smith, *Sensing the Past: Seeing, Hearing, Smelling, Tasting, and Touching in History* (California: University of California Press, 2007); Mark Michael Smith, ‘Producing Sense, Consuming Sense, Making Sense: Perils and Prospects for Sensory History’, *Journal of Social History*, 2007, 40, 841–58; Robert Jütte (trans), *A History of the Senses. From Antiquity to Cyberspace*, 2nd edn (Cambridge: Polity Press, 2005). On medicine and the senses see: Faith Wallis, ‘Medicine and the Senses: Feeling the Pulse, Smelling the Plague, and Listening for the Cure’, in Richard G. Newhauser, ed, *A Cultural History of the Senses in the Middle Ages* (London: Bloomsbury Academic, 2016), 133–52; William F. Bynum and Roy Porter, eds, *Medicine and the Five Senses*, 2nd edn (Cambridge: Cambridge University Press, 2004).

17 Michael Stolberg, ‘Examining the Body, C. 1500–1750’, in Sarah Toulalan and Kate Fisher, eds, *The Routledge History of Sex and the Body* (London-New York: Routledge, 2013), 91–105, 92.

18
*Ibid*., 91.

19 Biblioteca Civica *Girolamo Tartarotti* of Rovereto (henceforth BCRo), fondo *Manoscritti* (henceforth *Manoscritti*), codice 24 (henceforth cod. 24), *Consulti medici e ricette (sec. XVI) di Francesco Partini* (henceforth *Consulti medici*), f. 55r.

20
*Ibid*., ff. 180v–181r. On the catarrh see also Michael Stolberg, *Experiencing Illness and the Sick Body in Early Modern Europe* (Basingstoke-New York: Palgrave Macmillan, 2011), 28, 95.

21 Mattioli studied in Padua where he obtained his academic qualification in 1523. Giuseppe Fabiani, *La vita di Pietro Andrea Mattioli raccolta dalle sue opere* (Siena: Tip. dell’ancora di G. Bargellini, 1872), 5. Partini attended the Paduan University but passed his final exam at the Collegio dei Medici in Venice. Richard Palmer, *The “Studio” of Venice and Its Graduates in the Sixteenth Century* (Padua-Trieste: Edizioni Lint, 1983), 43.

22 Pietro Andrea Mattioli, *Commentarii secundo aucti, in libros sex Pedacii Dioscoridis Anazarbei de medica materia* (Venetiis: in Officina Valgrisiana, 1560), ff. α1r-β3r, f. β1; Pietro Andrea Mattioli, *Apologia adversus Amathum Lusitanum cum Censura in ejusdem enarrationes* (Venetiis: ex Officina Valgrisiana, 1559), f. A2r-v.

23 Paula Findlen, ‘The Formation of a Scientific Community: Natural History in Sixteenth-Century Italy’, in Anthony Grafton and Nancy G. Siraisi, eds, *Natural Particulars: Nature and the Disciplines in Renaissance Europe* (Cambridge: MIT Press, 1999), 369–400, 374.

24 Girolamo Tartarotti, *Saggio della biblioteca tirolese o sia Notizie istoriche degli scrittori della provincia del Tirolo* (Rovereto: presso Pierantonio Berno Libraio, 1733), 32; Giangrisostomo Tovazzi, *Medicaeum Tridentinum, id est, Syllabus medicorum Civitatis ac Dioecesis Tridentinae* (Trento: Marietti, 1889), 29.

25 See the consultations addressed to Nicolò Madruzzo: BCRo, *Manoscritti*, cod. 24, *Consulti medici*, ff. 50r–54r, 55r–58v, 59r–v, 60r–61r, 61v–63r, 64v–68v.

26 Mattioli, *Commentarii secundo aucti*, f. β1.

27 Alfonso Cetto, ‘A proposito di un manoscritto di Mattioli esistente nella Biblioteca Comunale di Trento (Ms. 1795)’, *Studi Trentini di Scienze Storiche*, 1959, 38, 233–57, 240.

28 Findlen, ‘The Formation of a Scientific Community’, 374.

29 Cesare Preti, ‘Mattioli, Pietro Andrea’, in *Dizionario Biografico degli Italiani* (Roma: Istituto dell’Enciclopedia italiana, 2008), LXXII, 308–11, 310. Mattioli probably arrived at Prague in February 1555: see Battista De Toni, ed, *Un pugillo di lettere di Giovanni Odorico Melchiori trentino a Ulisse Aldrovandi* (Venezia: Premiate officine grafiche C. Ferrari, 1925), 13.

30 Österreichische Staatsarchiv (henceforth AT-OeStA), Allgemeines Verwaltungsarchiv (henceforth AVA), Adelsarchiv, Reichsadelsakten (henceforth RAA), Allgemeine Reihe, 265.15.

31 On Mattioli see: Luca Ciancio, *Many Gardens—Real, Symbolic, Visual—of Pietro Andrea Mattioli*, in Juliette Ferdinand, ed, *From Art to Science. Experiencing Nature in the European Garden 1500-1700* (Treviso: ZeL Edizioni, 2016); Luca Ciancio, ‘*Per questa via s’ascende a magior seggio*. Pietro Andrea Mattioli e le scienze mediche e naturali alla corte di Bernardo Cles’, *Studi Trentini. Storia*, 2015, 94, 159–84; Daniela Fausti, ed, *La complessa scienza dei semplici. Atti delle celebrazioni per il V centenario della nascita di Pietro Andrea Mattioli (Siena, 12 marzo-19 novembre 2001)* (Siena: Accademia dei Fisiocritici, 2001); Findlen, ‘The Formation of a Scientific Community’; Findlen, ‘Courting Nature’, in Nicholas Jardine, James A. Secord, and Emma C. Spary, eds, *Cultures of Natural History* (Cambridge: Cambridge University Press, 1996), 57–74; Sara Ferri, ed, *Pietro Andrea Mattioli Siena 1501–Trento 1578. La vita le opere con l’identificazione delle piante* (Ponte S. Giovanni, PG: Quattroemme, 1997); Richard Palmer, ‘Medical Botany in Northern Italy in the Renaissance’, *Journal of the Royal Society of Medicine*, 1985, 78, 149–57.

32 Enrica Chiaramonte and Silvia Tozzi, ‘Un medico umanista fra dottrina e pratica’, in Ferri, ed, *Pietro Andrea Mattioli*, 61–81, 61.

33 Ciancio, ‘*Per questa via s’ascende a magior seggio’*, 164.

34 Lorenz Scholz, ed, *Consiliorum medicinalium, conscriptorum a praestantiss. atque exercitatiss. nostrorum temporum medicis liber singularis* (Hanoviae: Typis Wechelianis apud haeredes Ioannis Aubrii, 1610), 182, 220.

35 Österreichische Nationalbibliothek, Sammlung für *Handschriften und Nachlässe*, codex (henceforth cod.) 11182 Han, *Consilium datum in morbo Bohuslai cuiusdam …*, ff. 139r–77v; *ibid*., cod. 11155 Han, *Consilium pro Serenissimo Archiduce Ferdinando habitum ac decretum per collegium Domini Doctoris Iulii Alexandrini, Domini Doctoris Aipergeri, et Doctoris Matthioli*, ff. 27r–35v.

36 Tartarotti, *Saggio della biblioteca tirolese*, 115.

37 AT-OeStA, AVA, Adelsarchiv, RAA, Allgemeine Reihe, 307.50.

38 Georg Welsch, *Curationum Exotericarum Chiliades II. et Consiliorum Medicinalium Centuraie IV* (Ulmae: ex typographeo Christiani Balthas Kuenii Bibliopolae, 1676), 214–16.

39 BCRo, *Manoscritti*, cod. 24, *Consulti medici*.

40 Edoardo Benvenuti, ed, *I manoscritti della Biblioteca Civica di Rovereto descritti. Parte I ('300, ‘400, ’500, '600)* (Rovereto: Tipografia roveretana, 1908), 39.

41 BCRo, *Manoscritti*, cod. 24, *Consulti medici*, ff. 60r–61r.

42
*Ibid*., f. 61r.

43 Murphy, ‘Common Places’, 255–56.

44 Kassell, ‘Casebooks’, 602–3.

45
*Ibid*., 600, 624.

46 Stolberg, ‘A Sixteenth-century Physician’, 228, 230.

47 Stolberg, ‘Empiricism’, 494–96.

48 See the following consultations: BCRo, *Manoscritti*, cod. 24, *Consulti medici*, respectively ff. 31v–36r; 43v–49v; 60r–61r.

49 Pomata, ‘Sharing Cases’, 196.

50 Giovanni Battista Da Monte, *Consultationum medicarum opus absolutissimum* (Basel: [per Henricum Petri, et Petrum Pernam], 1565), 970, 981.

51 Murphy, ‘Common Places’, 261, 263.

52 BCRo, *Manoscritti*, cod. 24, *Consulti medici*, respectively ff. 23v–29r and 43v–49v; 50r–54r, 55r–58v, 59r–v, 60r–61r, 61v–63r, 64v–68v.

53
*Ibid*., f. 72r–v.

54
*Ibid*., ff. 31v–36v; 69r–71v; 74r–76v.

55
*Ibid*., ff. 73r–v; 164r–165v.

56
*Ibid*., ff. 86v–87v; 88r; 88v–89v; 96r–v.

57
*Ibid*., f. 135r.

58
*Ibid*., ff. 180v–183r.

59
*Ibid*., ff. 155r–156v.

60 Pietro Andrea Mattioli, *De morbi gallici curandi ratione*, in Pietro Andrea Mattioli *et al.*, *Morbi Gallici curandi ratio exquisitissima, a variis iisdemque peritissimis medicis conscripta. […]* (Lugduni: [expensis Scipionis de Gabiano et fratris], 1536), 47. On syphilis and its transmission and treatment modes, see Claudia Stein, *Negotiating the French Pox in Early Modern Germany* (Farnham: Ashgate, 2009); Concetta Pennuto, *Simpatia, fantasia e contagio. Il pensiero medico e il pensiero filosofico di Girolamo Fracastoro* (Roma: Edizioni di Storia e Letteratura, 2008); Kevin Brown, *The Pox. The Life and Near Death of a Very Social Disease* (Stroud, Gloucestershire: History Press, 2006); John Herderson, ‘Fracastoro, il legno santo e la cura del *mal francese*’, in Alessandro Pastore and Enrico Peruzzi, eds, *Girolamo Fracastoro fra medicina, filosofia e scienze della natura*. *Atti del Convegno internazionale di studi in occasione del 450^0^ anniversario della morte (Verona-Padua, 9-11 October 2003)* (Florence: Olschki, 2006), 73–89.

61 BCRo, *Manoscritti*, cod. 24, *Consulti medici*, f. 32r.

62
*Ibid.*, f. 59r.

63 ÖNB, *Handschriften*, cod. 11182 Han, *Consilium datum in morbo Bohuslai cuiusdam […]*, ff. 139r–177v.

64
*Ibid.*, f. 139v.

65
*Ibid.*, ff. 139v–140r.

66
*Ibid.*, f. 140r–v.

67
*Ibid.*, f. 140v.

68
*Ibid.*, f. 141r–v.

69 Pomata, ‘*Praxis historialis*’, 122; Esther Cohen, *The Modulated Scream: Pain in Late Medieval Culture* (Chicago: University Chicago Press, 2010), 100.

70 Taddeo Alderotti was an exponent of the new medical culture developed in the thirteenth century in opposition to those who considered medicine as a mere *ars mechanica*. Physicians, especially the ones from Bologna’s *Studium*, began to claim the status of *scientia* (learned discipline) for medicine. In their opinion, it had a theoretical basis and was connected with other branches of knowledge, above all natural philosophy. Nancy G. Siraisi, *Taddeo Alderotti and His Pupils. Two Generations of Italian Medical Learning* (Princeton: Princeton University Press, 1981), 119–20.

71 Teodoro Katinis, *Medicina e Filosofia in Marsilio Ficino. Il* consilio *contro la* pestilentia (Roma: Edizioni di Storia e Letteratura, 2007), 29; Piero P. Giorgi and Gian Franco Pasini, eds, Consilia *di Taddeo Alderotti XIII secolo* (Bologna: Istituto per la storia dell'Università di Bologna, 1997), 1, 5–6. However, Nancy Siraisi specifies that it is unlikely that Alderotti invented the genre of *consilia* in medieval medical writing (Michael Scot produced at least one similar description of an individual case for which he had been consulted while living in Bologna in 1220). It is true, however, that the earliest medieval *consilia* to survive, regardless of quantity, appear to be those emanating from Taddeo’s circle. Siraisi, *Taddeo Alderotti and His Pupils*, 270–71.

72
*Ibid*., 271–72.

73 ÖNB, *Handschriften*, cod. 11182 Han, *Consilium datum in morbo Bohuslai cuiusdam […]*, f. 143r–v.

74 Nancy G. Siraisi, ‘Giovanni Argenterio and Sixteenth-Century Medical Innovation. Between Princely Patronage and Academic Controversy’, *Osiris*, s. II, 1990, 6, 161–80: 170. For instance, the term ‘pleurisy’ indicated a general inflammation of the chest, but did not localise exactly which organ was affected. When diagnosing a subject with pleurisy, the physician did not exactly know which part was affected (pleura, lungs, heart or intercostal issue).

75 ÖNB, *Handschriften*, cod. 11182 Han, *Consilium datum in morbo Bohuslai cuiusdam […]*, ff. 146r, 147v.

76
*Ibid.*, f. 146r–v.

77
*Ibid.*, ff. 146v–147r.

78
*Ibid.*, f. 143r.

79 Chiara Crisciani, ‘Fatti, teorie, «narratio» e i malati a corte. Note su empirismo in medicina nel tardo medioevo’, *Quaderni storici*, 2001, 108, 695*–*717, 697–98.

80 See Tiziana Pesenti, *Professori e promotori di medicina nello Studio di Padova dal 1405 al 1509. Repertorio bio-bibliografico* (Padua–Trieste: Lint, 1984), 143–54.

81 Chiara Crisciani, ‘Medicine as Queen: The *Consilia* of Bartolomeo da Montagnana’, in Gideon Manning and Cynthia Klestinec, eds, *Professors, Physicians and Practices in the History of Medicine. Essays in Honor of Nancy Siraisi* (Cham: Springer, 2017), 88, 90.

82 Wallis, ‘Medicine and the Senses’, 141.

83
*Ibid*., 143.

84 Michael Stolberg, *Die Harnschau: eine Kultur- und Alltagsgeschichte* (Köln-Weimar: Böhlau, 2009),17–9.

85 Michael Stolberg, ‘The Decline of Uroscopy in Early Modern Learned Medicine (1500-1650)’, *Early Science and Medicine*, 2007, 12, 313–36, 315.

86 ÖNB, *Handschriften*, cod. 11182 Han, *Consilium datum in morbo Bohuslai cuiusdam […]*, ff. 156v-157r.

87 Michael Forcher, *Erzherzog Ferdinand II. Landesfürst von Tirol. Sein Leben, seine Herrschaft, sein Land* (Innsbruck: Haymon Verlag, 2017), 36.

88 BCRo, cod. 24, *Consulti medici e ricette*, ff. 180v–181r.

89
*Ibid*., f. 180v.

90
*Ibid.*, f. 181r.

91
*Ibid*.

92
*Ibid.*, f. 180v.

93 Giorgi and Pasini, eds, Consilia *di Taddeo Alderotti*, 194.

94 Georg Sarton, *Introduction to the History of Science*, 3 vols (Baltimore: Williams and Wilkins Company for Carnegie Institution of Washington, 1947), III, 1196. On Antonio Cermisone see also Paul F. Grendler, *The Universities of the Italian Renaissance* (Baltimore–London: The Johns Hopkins University Press, 2011), 24–5.

95 Antonio Cermisone, *Consilia medica* (Brixia: [Henricus de Colonia], 1476), f. 6v.

96 BCRo, *Manoscritti*, cod. 24, *Consulti medici*, ff. 180v, 181r.

97
*Ibid*., f. 180v.

98
*Ibid*., f. 180v.

99
*Ibid*., f. 181r.

100
*Ibid*., f. 182r.

101 Angelo Brumana, *‘Si servendum est, principibus serviendum.* Medici bresciani alla corte degli Asburgo nel XVI secolo’, *Misinta*, 2015, 43, 41–50, 49.

102 BCRo, *Manoscritti*, cod. 24, *Consulti medici*, f. 181v.

103
*Ibid*., ff. 180v, 181v.

104 Crisciani, ‘Medicine as Queen’, 88, 90.

105 BCRo, *Manoscritti*, cod. 24, *Consulti medici*, f. 182r.

106 Georg Melich, *Avvertimenti nelle compositioni de’ Medicamenti per uso della spetiaria, con una diligente esaminatione di molti simplici …* (in Venetia: appresso Giacomo Vincenti, 1605), 124r-v.

107 BCRo, *Manoscritti*, cod. 24, *Consulti medici*, ff. 180v, 181v.

108 Abram Smythe Palmer, *Folk-Etymology of Verbal Corruptions and Words Perverted in Form or Meaning, by False Derivation or Mistaken Analogy* (New York: Haskell House Publishers Ltd., 1969), 220.

109 Giacomo Rossetto, *I libri di Giovanni Mesue dei semplici purgativi, Et delle medicine composte …* (Venetiis: ex bibliotheca aldina, 1589), 254.

110 BCRo, *Manoscritti*, cod. 24, *Consulti medici*, f. 180v.

111 Pietro Andrea Mattioli, *I discorsi nei sei libri della materia medicinale di P. Dioscoride …* (in Venetia: appresso Vincenzo Valgrisi, 1563), 641.

112 BCRo, *Manoscritti*, cod. 24, *Consulti medici*, f. 181v.

113
*Ibid*., f. 182v.

114
*Ibid*., f. 182r.

115 Giorgi and Pasini, eds, Consilia *di Taddeo Alderotti*, 195.

116
*Ibid*., 195–99.The ‘Six Non-Natural Things’ represent the main focus of the monograph by Sandra Cavallo and Tessa Storey, which constitutes the fullest discussion of this topic in English. See Sandra Cavallo and Tessa Storey, *Healthy Living in Late Renaissance Italy* (Oxford: Oxford University Press, 2013).

117 Giorgi and Pasini, eds, Consilia *di Taddeo Alderotti*, 124: ‘The cure by means of medicines is of the following kind: among medicines, some are taken internally; some are applied externally. Some of those which are taken internally are purgative medicines; some are alter and soothe. Among medicines which are taken internally, one has to deal with purgative medicines first. Furthermore, the evacuation is double: one is certainly universal, the other is particular’.

118
*Ibid*., 129.

119 ÖNB, *Handschriften*, cod. 11155 Han, *Consilium pro Serenissimo Archiduce Ferdinando habitum ac decretum per collegium Domini Doctoris Iulii Alexandrini, Domini Doctoris Aipergeri, et Doctoris Matthioli*, ff. 27r–35v.

120 Heinrich Hermelink, ed, *Die Matrikeln der Universität Tübingen*, 3 vols (Stuttgart: Druck und Verlag von W. Kohlhammer, 1906), I, 365. On Heyperger’s age see the *Stammbuch* by Johann Valentin Deyger, kept at the Tübingen University Library, Mh 1030, f. 168. The *quaestio* (and the relevant *positiones*) discussed by Heyperger during his thesis defense have been published online by the Munich Ludwig-Maximilians-Universität: https://epub.ub.uni-muenchen.de/16166/1/W2H.lit.176_76.pdf

121 ÖNB, *Handschriften*, cod. 11155 Han, *Pro Serenissimo et Potentissimo Archiduce Ferdinando Austriaco Consilium Renati Brasavoli Ferrariensis Medici*, f. 24v.

122 Alfonso Lazzari, *Un umanista romagnolo alla corte d’Ercole II d’Este: Bartolomeo Ricci da Lugo* (Ferrara: G. Zuffi, 1903), 160; Giuseppe Pardi, *Lo Studio di Ferrara nei secoli XV e XVI con documenti inediti* (Ferrara: G. Zuffi, 1903), 232.

123 Giuliano Gliozzi, ‘Brasavola (Brasavoli) Antonio, detto Antonio Musa’, in *Dizionario Biografico degli Italiani* (Roma: Istituto dell’Enciclopedia italiana, 1972), XIV, 51–53, 51.

124 ÖNB, *Handschriften*, cod. 11155 Han, *Pro Serenissimo et Potentissimo Archiduce Ferdinando*, f. 2v.

125
*Ibid*., *Pro Serenissimo Archiduce Ferdinando*, f. 28r.

126
*Ibid*.: ‘The air in this place is also notably changeable. In fact, it occurs that in one and same day, and sometimes even in one and same hour, the air gets warm and cools down drastically. Thing which not only demolishes ill bodies and its parts subject to diseases, but also affects healthy and strong ones very much’.

127
*Ibid*.

128
*Ibid*., f. 28v.

129
*Ibid*.

130
*Ibid*., f. 30r.

131
*Ibid.*, f. 34r.

132 Antonio Targioni Tozzetti, *Corso di botanica medico-farmaceutica e di materia medica* (Florence: per Vincenzo Batelli e compagni, 1847), 344.

133 ÖNB, *Handschriften*, cod. 1155 Han, *Pro Serenissimo Archiduce Ferdinando*, f. 35r.

134 Nenter Georges Philippe, Stahl Georg Ernst, *Fundamenta medicinae theoretico-practica* (Venetiis: apud Sebastianum Coleti, 1735), 65.

135 Joseph Jacob von Plenck, *Tossicologia ossia dottrina intorno i veleni e i loro antidoti … tradotta dall’originale latino in italiano* (Naples: presso Giuseppe Maria Porcelli Libraio e Stampatore della R. Acc. Militare, 1790), 169.

136 Mary Lindemann, *Medicine and Society in Early Modern Europe. New Approaches to European History* (Cambridge: Cambridge University Press, 1999), 238, 273–75.

137 Richard Palmer, ‘In Bad Odour: Smell and its Significance in Medicine from Antiquity to the Seventeenth Century’, in Bynum and Porter, eds, *Medicine and the Five Senses*, 61–68, 63–64.

138 Stolberg, ‘Bedside Teaching’, 648, 651.

139 Stolberg, ‘Examining the body’, 92.

140 See ÖNB, *Handschriften*, cod. 11182 Han, *Consilium datum in morbo Bohuslai cuiusdam […]*, f. 142r.

141 Hannah Murphy, *A New Order of Medicine: The Rise of Physicians in Reformation Nuremberg* (Pittsburgh: University of Pittsburgh Press, 2019), 4–6.

142 Stefania Fortuna, ‘Galeno e le sue traduzioni’, *I Quaderni del Ramo d’Oro on-line*, 2012, 5, 112–22, 115. On the same topic see also Fortuna, ‘Editions and Translations of Galen from 1490 to 1540’, in Petros Bouras-Vallianatos and Barbara Zipser, eds, *Brill’s Companion to the Reception of Galen* (Leiden–Boston: Brill, 2019), 437–52.

143 Andrea Carlino, *Anatomia umanistica: Vesalio, gli Infiammati e le arti del discorso*, in Maria Conforti, Andrea Carlino, and Antonio Clericuzio, eds, *Interpretare e curare. Medicina e salute nel Rinascimento* (Roma: Carocci, 2013), 77–94, 81.

144 Vivian Nutton, ‘Renaissance Galenism, 1540–1640: Flexibility or an Increasing Irrelevance?’, in Bouras-Vallianatos and Zipser, eds, *Brill’s Companion*, 472–86, 472–73.

145 Nancy Siraisi, ‘Giovanni Argenterio and Sixteenth-Century Medical Innovation. Between Princely Patronage and Academic Controversy’, *Osiris*, s. II, 1990, 6, 161–80, 169.

146 Concetta Pennuto, ‘La natura dei contagi in Fracastoro’, in Pastore and Peruzzi, eds, *Girolamo Fracastoro*, 57–72, 64, 71.

147 Cavallo and Storey, *Healthy Living*, 6.

148 Andrea Gallo, *Fascis de peste* (Brixiae: ex officina Io. Baptistae Bozolae, 1565). On the frontispiece of the volume, the professional titles of Gallo are spelled out: ‘Physician of Emperor Ferdinand and archiater of Archduke Ferdinand’. Though Gallo’s death date is unknown, by 1565 he was dead, and his work was published by his sons Giulio, Guglielmo, and Ludovico. These latter addressed the dedicatory letter of the work to Archduke Ferdinand, in which they reported that, since Andrea had been seized by mistimed death, he had not been able to finish his work. Though incomplete, his sons decided to publish it, since it could help physicians treat the plague. Gallo, *Fascis*, f. 2r.

149 Ciancio, ‘*Per questa via s’ascende a magior seggio’*, 164.

150 Richard Palmer and Andreina Zitelli, ‘Le teorie mediche sulla peste e il contesto veneziano’, in Iid., eds, *Venezia e la Peste 1348-1797*, Catalogue of the Exhibition in Venice, Museum Correr (Venice: Marsilio, 1980), 21–8, 22–3.

151 Cavallo and Storey, *Healthy Living*, 145.

152 Pomata, ‘*Praxis historialis*’, 128.

153 BCRo, *Manoscritti*, cod. 24, *Consulti medici*, f. 181r.

154 ÖNB, *Handschriften*, cod. 11182 Han, f. 143r. See also Claudius Galenus, *De locorum affectorum notitia libri sex, Gulielmo Copo Basiliensi interprete …* (Lugduni: apud Gulielmum Rouillium, 1549), 330–31.

155 ÖNB, *Handschriften*, cod. 11182 Han, f. 146r.

156
*Ibid*., ff. 144v–146r.

157 BCRo, *Manoscritti*, cod. 24, *Consulti medici*, f. 182r.

158 See respectively BCRo, *Manoscritti*, cod. 24, *Consulti medici*, f. 182r; Giorgi and Pasini, eds, Consilia *di Taddeo Alderotti*, 125.

159 Crisciani, ‘Medicine as Queen’, 87.

160 Cohen, *The Modulated Scream*, 101.

161 Crisciani, ‘Fatti, teorie, ”narratio”’, 697.

## Notes

In 2016, Alessandra Quaranta was awarded a post-doctoral scholarship at the University of Trento. The results of the research conducted there in the field of the Early-Modern History of Medicine are published in a volume issued in 2019. Her doctoral thesis, entitled *Medici italiani eretici*, was published in the same year. Since October 2020, she has been a post-doctoral researcher at the Institute of History of the University of Vienna, Austria.

